# A Rare Acute Complication Early After Glucocorticoid Administration in a Case of Autoimmune Haemolytic Anemia (AIHA): A Case Report

**DOI:** 10.7759/cureus.89038

**Published:** 2025-07-30

**Authors:** Raaj Pawan Kumar Lingamgunta, Naga Sai Gouthami Gurujala, Abhishek Perumallapalli

**Affiliations:** 1 Internal Medicine, NRI Medical College, Chinakakani, IND; 2 General Medicine, NRI Medical College, Chinakakani, IND

**Keywords:** an unusual case, autoimmune hemolytic anemia (aiha), deep vein thrombosis (dvt), systemic steroid therapy, thrombosis

## Abstract

Pulmonary embolism (PE) and deep vein thrombosis (DVT) are well-known lethal complications in autoimmune hemolytic anemia (AIHA). Here, we describe a case of a 45-year-old male patient with AIHA who developed DVT early after glucocorticoid administration. The patient presented with severe anemia, suggesting active hemolysis.

Deep vein thrombosis was confirmed within two weeks after glucocorticoid administration, suggesting that both active hemolysis and glucocorticoid administration synergistically contributed to the development of DVT. Clinicians should consider that such synergism may increase the risk of thromboembolism in patients with AIHA, and prophylactic anticoagulants are worth considering in these patients after starting glucocorticoids.

## Introduction

Autoimmune hemolytic anemia (AIHA) is a rare condition where the body’s immune system attacks its own red blood cells causing hemolysis [1]. AIHA includes warm AIHA, cold agglutinin disease (CAD), mixed AIHA, paroxysmal nocturnal hemoglobinuria and atypical AIHA [2]. Most AIHA cases are of the warm type, in which immunoglobulin G (IgG) autoantibodies are formed against red blood cell (RBC) surface antigens. Autoantibody-coated RBCs pass through the spleen, where splenic macrophages recognise and destroy them causing extravascular hemolysis. Intravascular hemolysis occurs due to complement activation in half of patients with AIHA [3].

AIHA is associated with an increased risk of thromboembolism [3]. It was observed that approximately 11.5% of patients with AIHA develop thromboembolic events, highlighting the significant thrombotic risk in this population. Moreover, corticosteroid therapy has been independently linked to an increased risk of venous thromboembolism, thereby further elevating the overall thrombotic risk. Several mechanisms for thromboembolism development in AIHA patients have been proposed. These include phosphatidylserine exposure, free hemoglobin and heme release, microvascular shedding, erythrocyte ADP and erythrocyte arginase. These factors are associated with higher thrombosis incidence [4]. In addition, glucocorticoid administration can increase the risk of thrombosis by promoting endothelial dysfunction and platelet activation [5].

Here, we describe a patient diagnosed as AIHA who developed thrombosis early after starting systematic glucocorticoids.

## Case presentation

A 45-year-old male patient presented to the hospital with chief complaints of yellowish discoloration of urine, fever, and shortness of breath for 10 days. On examination, he was found to have pallor and splenomegaly. The blood investigation is given in Table 1.

**Table 1:** Complete Blood Count RBC count: Red blood cell count; WBC count: White blood cell count; PCV: Packed cell volume; MCV: Mean corpuscular volume; MCH: Mean corpuscular hemoglobin; MCHC: Mean corpuscular hemoglobin concentration; RDW: Red cell distribution width.

Complete blood count	Results	Normal values
Hemoglobin	2.6 g/dL	13.5 - 17.5 g/dL (males)
RBC count	0.74 million/mm^3^	4.5 - 5.9 million/mm^3^
WBC count	7100/mm^3^	4000 - 11000/mm^3^
PCV	7.7%	41 - 53% (males)
MCV	104.1 fL	80 - 100 fL
MCH	35.1 pg	27 - 33 pg
MCHC	33.8 g/dL	32 - 36 g/dL
RDW	21.3%	11.5 - 14.5%
Reticulate count	5%	0.5 - 2.5%

The patient demonstrated severe anemia with a hemoglobin level of 2.6 g/dl, markedly below the normal range (13.5-17.5 g/dl). RBC count was critically low at 0.74 million/mm^3^, and packed cell volume (PCV) was reduced to 7.7%. The mean corpuscular volume (MCV) was 104.1 fL, indicating macrocytic anemia. Mean corpuscular hemoglobin​​​​​​​ (MCH) and mean corpuscular hemoglobin concentration​​​​​​​ (MCHC) were elevated at 35.1 pg and 33.8 g/dl, respectively, while red cell distribution width​​​​​​​ (RDW) was increased to 21.3%, suggesting anisopoikilocytosis. A reticulocyte count of 5% pointed towards a regenerative marrow response, often seen in hemolytic or blood loss anemia. The renal function test result is given in Table 2.

**Table 2:** Renal function test parameters S. urea: Serum urea; S. creatinine: Serum creatinine; S. sodium: Serum sodium; S. potassium: Serum potassium

Renal function test	Result	Normal range
S. urea	61 mg/dL	10 - 40 mg/dL
S. creatinine	1.0 mg/dL	0.6 - 1.2 mg/dL
S. sodium	134 mmol/L	135 - 145 mmol/L
S. potassium	4.1 mmol/L	3.5 - 5.0 mmol/L

Renal parameters are serum creatinine at 1.0 mg/dL, sodium at 134 mmol/L, and potassium at 4.1 mmol/L, within normal limits but mildly elevated urea at 61 mg/dL, possibly due to catabolic state or dehydration. The liver function test result is given in Table 3.

**Table 3:** Liver function test parameters AST: Aspartate Aminotransferase; ALT: Alanine Aminotransferase; S. protein: Serum protein; S. albumin: Serum albumin; S. globulin: Serum globulin; A/G RATIO: Albumin/Globulin ratio.

Liver function test	Result	Normal range
Total Bilirubin	4.6 mg/dL	0.3 - 1.2 mg/dL
Direct Bilirubin	0.4 mg/dL	0 - 0.3 mg/dL
Indirect bilirubin	4.2 mg/dL	0.2 - 0.8 mg/dL
AST	37 IU/L	10 - 40 IU/L
ALT	32 IU/L	7 - 56 IU/L
S. protein	4.2 g/dL	6.4 - 8.3 g/dL
S. albumin	2.3 g/dL	3.5 - 5.0 g/dL
S. globulin	1.9 g/dL	2.3 - 3.5 g/dL
Alkaline phosphatase	68 IU/L	38 - 126 IU/L
A/G RATIO	1.5	1.0 - 2.1

Liver function analysis revealed a total bilirubin level of 4.6 mg/dL, with a direct fraction of 0.4 mg/dL and a markedly elevated indirect bilirubin of 4.2 mg/dL, consistent with unconjugated hyperbilirubinemia typically seen in hemolytic anemia. Liver enzymes were within or slightly above normal limits: Alanine aminotransferase (ALT) at 32 IU/L and aspartate aminotransferase (AST) at 37 IU/L, while alkaline phosphatase was 68 IU/L. Serum albumin was reduced to 2.3 g/dL, and globulin was 1.9 g/dL, resulting in a total protein of 4.2 g/dL. The A/G ratio was 1.5, within normal range. These findings do not suggest intrinsic liver pathology, but rather reflect hemolytic jaundice due to increased breakdown of red cells. The additional diagnostic tests result is given in Table 4.

**Table 4:** Additional diagnostic tests LDH: Lactate dehydrogenase; S. folate: Serum folate; S. vitamin B_12_: Serum vitamin B_12_

Additional diagnostic tests	Result	Normal range
LDH	1077 IU/L	140 - 280 IU/L
S. folate	5.49 ng/mL	3 - 17 ng/mL
S. vitamin B12	> 2000 pg/mL	200 - 900 pg/mL

Serum folate was within normal range, while vitamin B12 was markedly elevated (>2000 pg/mL), possibly due to supplementation or underlying hematological stress. Lactate dehydrogenase (LDH) was markedly elevated at 1077 IU/L, supporting increased cell turnover or hemolysis. The anemia profile is given in Table 5.

**Table 5:** Anemia profile S. iron: Serum iron; TIBC: Total Iron Binding Capacity; S. ferritin: Serum ferritin

Anemia profile	Result	Normal range
S. Iron	233 μg/dL	60 - 70 μg/dL
TIBC	287.3 μg/dL	240 - 450 μg/dL
S. ferritin	912 ng/mL	24 - 336 ng/mL

Iron studies revealed normal serum iron (233 μg/dL) and total iron binding capacity (TIBC) (287.3 μg/dL) with elevated ferritin (912 ng/mL), indicating adequate iron stores, thereby ruling out iron deficiency anemia. The Coombs test result is given in Table 6.

**Table 6:** Coombs test results DCT: Direct Coombs Test; ICT: Indirect Coombs Test

Coombs test	Result	Normal range
DCT	4+	Negative
ICT	3+	Negative

Both direct and indirect Coombs tests were positive (4+ and 3+, respectively), strongly suggesting an autoimmune hemolytic anemia (AIHA).

Peripheral blood smear showed reduced RBC density with anisopoikilocytosis, presence of microcytes, tear drop cells, elliptocytes, and occasional nucleated RBCs.

Based on these findings, he was diagnosed with Coombs-positive AIHA. He was started on IV inj. Methylprednisolone 1 gm for three days, and three doses were given. Since there was no response, the first dose of Rituximab was given on the third day of Methylprednisolone administration, after medical oncology consultation. IV inj. Methylprednisolone 1 gm was continued for two more days and was followed by a maintenance steroid dose of 50 mg PO OD (1 mg/kg) of prednisolone.

**Figure 1:**
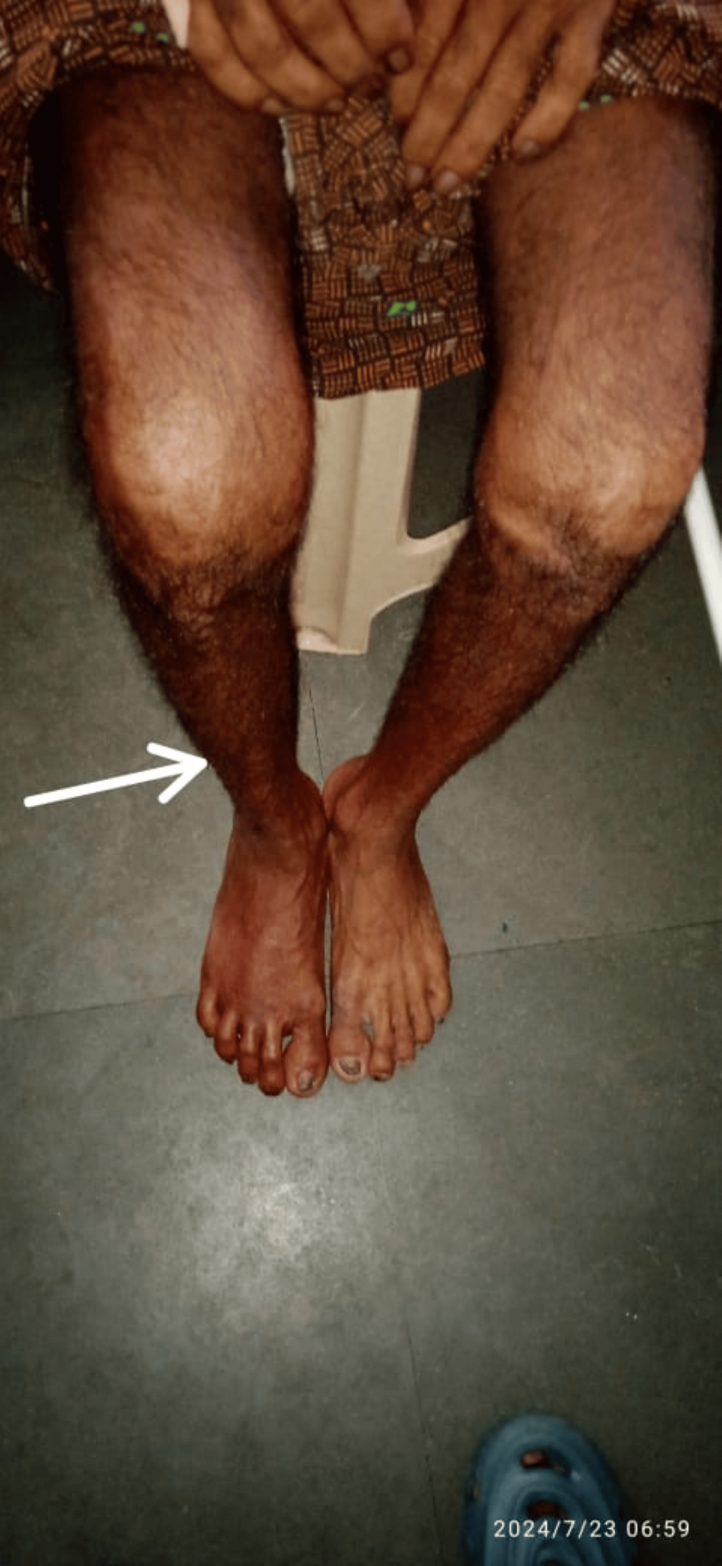
Swelling and Cyanotic Discoloration of the Left and Right Lower Limb in Deep Vein Thrombosis

The second dose of Rituximab was given one week after the first dose. During this period, the patient was transfused with 3 units of packed RBCs, which resulted in increased hemoglobin to 11 g/dl. When the patient was advised to follow up for the third dose of Rituximab, the patient complained of bilateral lower limb calf muscle pain while walking. Clinical examination revealed swelling and cyanotic discolouration of both lower limbs (Figure 1).

**Figure 2:**
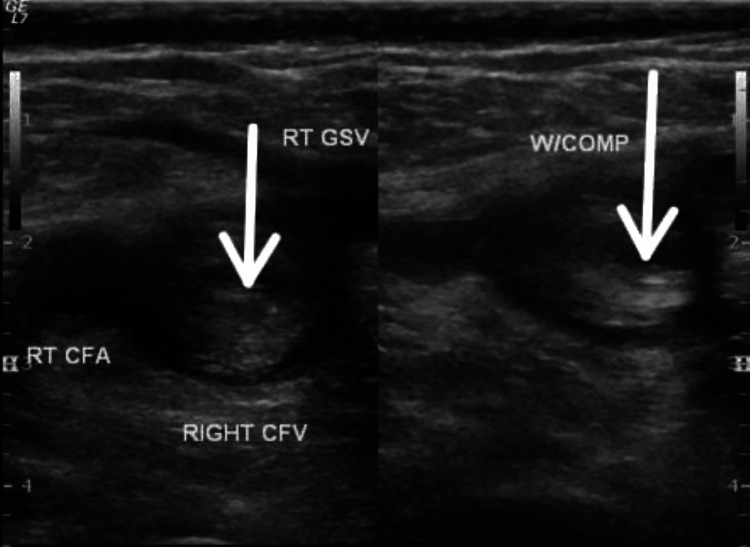
Right - Non-Compressible CFV, Left - Echogenic Thrombus Within CFV RT CFA: Right common femoral artery; RT GSV: Right great saphenous vein; CFV: Common femoral vein; W/COMP: with compression.

A venous Doppler was performed, which showed acute bilateral deep vein thrombosis (DVT) involving the common femoral veins (Figure 2).

To evaluate the cause of DVT, a thrombophilia workup was performed. Results showed levels of Protein C, Protein S, Antithrombin III, and homocysteine were within normal limits. Tests for Factor V Leiden mutation and lupus anticoagulant were negative. These findings excluded underlying inherited or acquired thrombophilic conditions.

Importantly, the patient had no recent trauma, immobilisation, surgery, or prolonged bed rest. He remained ambulatory during most of the hospital stay. Therefore, the DVT was attributed to the synergistic effect of active hemolysis and initiation of high-dose corticosteroid therapy.

The patient was initiated on IV unfractionated heparin and was continued with direct oral anticoagulants (Tab. Apixaban) along with maintenance steroids. Follow-up venous Doppler imaging was performed after three weeks of anticoagulation therapy, which showed complete resolution of thrombosis in the common femoral vein. Clinically, the patient showed improvement with resolution of calf pain and normalisation of limb color and temperature. Then, on the next follow-up, the third and fourth doses of Rituximab were completed without further issues.

A clinical timeline was constructed as follows:

Day 1: Diagnosis of AIHA + Inj. Methylprednisolone 1gm (1st dose) + one unit of packed red blood cells (PRBCs) (1st unit)

Day 2: 2nd dose of IV Methylprednisolone 1g + one unit of PRBCs (2nd unit) administered

Day 3: 3rd dose of IV Methylprednisolone 1g + one unit of PRBCs (3rd unit) + Rituximab (1st dose)+ Repeated Hb level = 4 gm/dl

Day 4: 4th dose of IV Methylprednisolone 1g + Repeated Hb level = 5.4 gm/dl

Day 5: 5th dose of IV Methylprednisolone 1g + Repeated Hb level = 8.8 gm/dl

From Day 6: Maintenance steroid dose of 50mg PO OD of prednisone

Day 10: 2nd dose of Rituximab + Repeated Hb level = 11 gm/dl

Day 17: Advised for 3rd dose of Rituximab, but diagnosed with DVT + IV unfractionated heparin given + continued with Tab. Apixaban

After three weeks: Follow-up venous Doppler done, revealed complete resolution of DVT.

Day 38: 3rd dose of Rituximab given

Day 45: 4th dose of Rituximab given

## Discussion

Autoimmune hemolytic anemia is a rare medical condition, which delineates immune-mediated destruction of red blood cells. While the main objective is on anemia and hemolysis, accumulating evidence suggests a strong association between AIHA and thrombotic events such as Deep Vein Thrombosis (DVT) and Pulmonary Embolism (PE) [6,7].

Hemolysis in AIHA leads to a hypercoagulable state through multiple mechanisms. Free hemoglobin is released into the circulation and binds to nitric oxide, resulting in vasoconstriction, platelet aggregation and endothelial damage [8]. In addition to that, inflammatory cytokines and cell-free DNA are released from damaged cells, which could further worsen thrombus formation [9].

Corticosteroids play a vital role in the management of AIHA. Although steroids are effective in controlling hemolysis, they are also known to promote prothrombotic states by inducing insulin resistance, dyslipidemia, hypertension and coagulation factors imbalance [10]. The possibility of thrombosis is especially high during the initial weeks following steroid initiation [7,11].

Even with these known associations, at present, there are no globally accepted guidelines for prophylactic anticoagulation in AIHA patients. Some experts suggest thromboprophylaxis, specially in hospitalized or high-risk patients, but this application needs to be balanced against bleeding risks [11,12].

Various case reports and observational studies have emphasized DVT and PE as serious complications in AIHA [6,8,13]. A recent retrospective review stressed that multi-treatment regimens and intravascular haemolysis increase the possibility of thrombotic events [13]. Furthermore, mixed forms of AIHA or those refractory to steroids may need more assertive immunosuppressive therapy, worsening the risk-reward ratio for anticoagulation [14].

Our case focuses on the need for increased understanding of thromboembolic risk in AIHA, particularly following steroid initiation. Appropriate recognition and intervention are crucial, as untreated DVT can result in lethal effects such as pulmonary embolism [8,12,14,15]. More reliable clinical trials are required to develop risk assessment tools and thromboprophylaxis guidelines for AIHA patients.

## Conclusions

This case highlights an unacknowledged complication of thromboembolism in a patient with autoimmune hemolytic anemia, especially after initiating corticosteroid therapy. Although steroids remain as the mainstay of treatment in managing hemolysis, their prothrombotic effects possibly will increase the risk of deep vein thrombosis. Clinicians should maintain a high index of suspicion for thrombotic complications in AIHA, particularly after initiating corticosteroid therapy. Early identification and suitable management of thrombosis can remarkably reduce morbidity and anticipate serious outcomes. Our report highlights the need for apparent guidelines on risk assessment and prophylactic anticoagulation in AIHA patients.
